# Model-Assisted Analysis of Spatial and Temporal Variations in Fruit Temperature and Transpiration Highlighting the Role of Fruit Development

**DOI:** 10.1371/journal.pone.0092532

**Published:** 2014-03-24

**Authors:** Thibault Nordey, Mathieu Léchaudel, Marc Saudreau, Jacques Joas, Michel Génard

**Affiliations:** 1 CIRAD, UPR HORTSYS, Saint-Pierre, La Réunion, France; 2 INRA, UMR 547 PIAF, BP 10448, Clermont-Ferrand, France; 3 CIRAD, UMR QUALISUD, Montpellier, France; 4 INRA, UR 1115, Plantes et Systèmes de Culture Horticoles, Avignon, France; Universidade Federal de Vicosa, Brazil

## Abstract

Fruit physiology is strongly affected by both fruit temperature and water losses through transpiration. Fruit temperature and its transpiration vary with environmental factors and fruit characteristics. In line with previous studies, measurements of physical and thermal fruit properties were found to significantly vary between fruit tissues and maturity stages. To study the impact of these variations on fruit temperature and transpiration, a modelling approach was used. A physical model was developed to predict the spatial and temporal variations of fruit temperature and transpiration according to the spatial and temporal variations of environmental factors and thermal and physical fruit properties. Model predictions compared well to temperature measurements on mango fruits, making it possible to accurately simulate the daily temperature variations of the sunny and shaded sides of fruits. Model simulations indicated that fruit development induced an increase in both the temperature gradient within the fruit and fruit water losses, mainly due to fruit expansion. However, the evolution of fruit characteristics has only a very slight impact on the average temperature and the transpiration per surface unit. The importance of temperature and transpiration gradients highlighted in this study made it necessary to take spatial and temporal variations of environmental factors and fruit characteristics into account to model fruit physiology.

## Introduction

Numerous physiological processes involved in fruit development depend on temperature. Temperature is implied in fruit growth [1]–[4], fruit respiration [5]–[7] and fruit ripening [8]. In addition, temperature has a major impact on fruit physiology through its effect on fruit water losses by transpiration [9], [10] due to its influence on the pressure vapour deficit (VPD) that drives the transpiration rate [11]. Water losses by transpiration are in fact responsible for fruit diurnal shrinkage [2], [12], and affect both growth rate [13], [14] and fruit quality [14], [15]. Many studies have highlighted the impact of temperature on several fruit quality traits such as appearance, taste and size [16]–[18].

The temperature of a fruit results from its heat budget, which is defined by energy exchanges caused by radiation, evaporation, convection, conduction and metabolic activity [19]. Different factors have an impact on the components of the fruit heat budget and can be broken down into environmental factors (solar radiation, air moisture, air temperature and wind), fruit thermal properties (heat capacity, density and conductivity), and fruit physical properties (skin permeability to water diffusion, peel reflectance and fruit volume) [20].

Modelling approaches based on physical processes made it possible to highlight the impact of variations in environmental factors on plant organ temperature, as shown on sunflower capitulum [21], maize ear [22] and fruit [20]. The model developed by Saudreau et al. [20] on peach and apple fruits revealed that the heterogeneity of environmental conditions at the fruit scale induced large temperature gradients within the fruit. However, until now, no model has focused on the effect of the variations of the fruit's physical and thermal properties induced by its development on the fruit temperature.

Many studies have shown that fruit properties involved in fruit temperature variation were modified by the fruit environment [18], [23]–[25] and during its development [26], [27]. Water content is known to be related to the three fruit thermal properties - conductivity, heat capacity [28] and specific gravity [27]. However, it was reported that water content changes during fruit development [2], [29] and depending on the fruit tissue (peel, pulp or stone) [30]. Furthermore, coloration of fruit skin is known to vary due to variations in the contents of peel pigments [31] as a result of fruit development and exposure to light [32]. Since pigment contents affect fruit reflectance [33], changes in peel colour are assumed to be related to variations in fruit optical properties involved in radiation flux [34]. Peel conductance to water, which regulates fruit transpiration, also varies with fruit development [26], [35] and sun exposure [25], [36].

The aim of this study was therefore to determine how changes in thermal and physical properties during fruit development affect spatio-temporal variations of the fruit's temperature and its transpiration.

A tropical fruit, the mango (cv. “Cogshall”), was taken as reference since it is a large-size drupe that grows under high-temperature environments.

First, changes in fruit density, heat capacity, thermal conductivity and size, as well as peel reflectance and conductance to water, were measured during fruit development in contrasting sun exposure conditions. A model to predict spatial and temporal variations of fruit transpiration and temperature was then developed, taking the heterogeneity and the evolution of fruit thermal and physical properties within the fruit tissues and during their development into account. After evaluating the quality of model predictions, the model was used to assess the effect of a fruit's thermal and physical properties on its temperature and transpiration.

## Materials and Methods

### Model presentation

**Figure 1:**
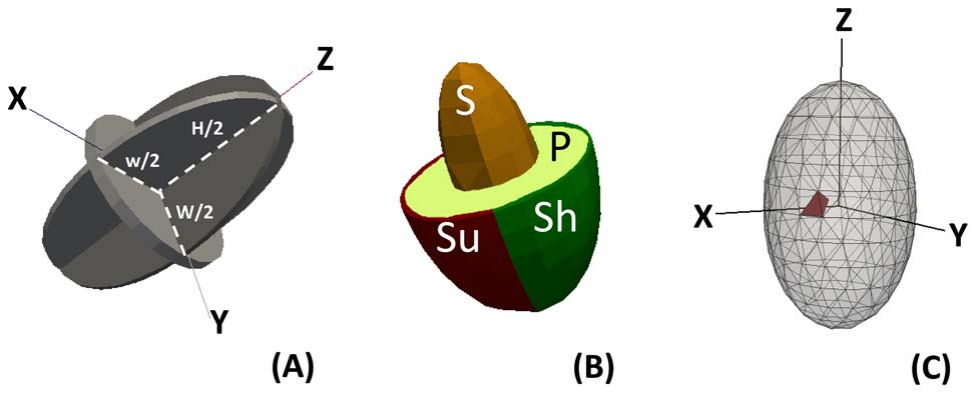
Schematic representation of the mango volume as an ellipsoid shape (A) defined by three distinct semi-axes, corresponding to its semi-length (H), semi-large diameter (W) and semi-small diameter (w); of the mango tissues (B), including three compartments, the stone (S), the pulp (P) and the peel, separated between the sunny (Su) and the shaded (Sh) sides; and of the fruit mesh and its sub-units (C).

The model presented simulates spatial and temporal variations of temperature and transpiration of a mango fruit during its development. Cogshall mango fruit and its stone were considered to have a triaxial ellipsoid shape defined by three distinct semi-axes: the length (H), the large diameter (W) and the small diameter (w) (Figure 1A). The fruit was considered to be located at the exterior of the canopy, with a sunny side directly exposed to sun radiation and with a shaded side that received a fraction of the global radiation. The simulated mango fruit was considered to be composed of four different tissues: the pulp, the stone, and the shaded and sunny parts of the peel, characterised by different physical and thermal properties (Figure 1B).

The temperature of a system is linked to its heat budget and varies with the gain or loss of energy. On the basis of the first law of thermodynamics without any energy loss due to work, the relationship between temperature variation (ΔT, in Kelvin) and heat variation (ΔQ, in Joules) is shown in Equation 1, where Cp is the heat capacity (in J.Kg^−1^.K^−1^), V is the volume of the tissue (in m^3^) and S_g_ is the specific gravity of the tissue (Kg.m^−3^):
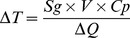
(1)


In the case of a whole fruit in a tree, the heat budget (ΔQ) varies as a result of (i) energy exchanges at the fruit surface by radiation, transpiration, conduction and convection, and (ii) the energy source within the fruit due to chemical activities [19]. The heat released from metabolic activities within the fruit and the energy exchanged between the plant and the fruit were assumed to be low and were therefore not taken into account.

ΔQ was determined using Equation 2, where the heat rate as a function of radiation (P_Radiation_), transpiration (P_Transpiration_), convection (P_Convection_) and conduction (P_Conduction_) is expressed in Watts, and the time (Δt) is expressed in seconds:
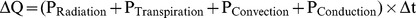
(2)


Heat flux equations are taken from the spatio-temporal model of fruit temperature developed by Saudreau et al. [20] and are summarised in the following sections.

#### Radiation

The radiation heat rate (P_Radiation_, W) received by a surface S (m^2^) was determined using Equation 3, where A_sw_ and A_lw_ are the fruit surface reflectance coefficients for short-wave and long-wave radiations, respectively, 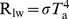 is the long-wave radiation component (TIR, in W.m^−2^) calculated with the air temperature (T_a_, K), R_sw_ is the short-wave radiation component (PAR and NIR, in W.m^−2^) of the global radiation, T_s_ (K) is the fruit surface temperature, and σ is the Stefan–Boltzmann constant ( = 5.67.10^−8^ J K^−4^ m^−2^ s^−1^).
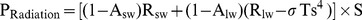
(3)


#### Transpiration

The latent heat rate released by transpiration (P_Transpiration_, W) through a surface S (m^2^) was calculated using Equation 4, as proposed by Monteith and Unsworth [11]:
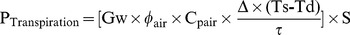
(4)where Gw (m.s^−1^) is the fruit surface conductance to water, 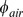
(Kg.m^−3^) is the air density estimated using equations established by Picard et al. [37], Cp_air_ is the air heat capacity considered as a constant  = 1004 J.Kg^−1^.K^−1^, τ is the psychometric constant (66.5 Pa.K^−1^), Td (K) is the temperature at the dew point, and Δ is the rate of increase in saturation vapour pressure with the temperature at the dew point, deduced from the relationship established by Buck [38]. The mass of water lost by transpiration (Kg) was deduced from the amount of energy (J) dissipated by transpiration by the whole fruit and the water enthalpy of vaporisation considered as a constant (2.25×10^6^ J.Kg^−1^).

#### Convection

The sensible heat rate by convection (P_Convection_, W) through a surface S (m^2^) was calculated using Equation 5. The value of the convective heat transfer coefficient (h, W.m^−2^.K^−1^) was calculated with relationships established for a spherical shape [39], since, to our knowledge, no relationship has yet been found for an ellipsoidal form. This hypothesis seems reasonable since drag forces are fairly similar in a vertical ellipsoid and in a sphere [40].
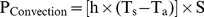
(5)


#### Conduction

The heat rate by conduction (P_Conduction_, W) through a surface S (m^2^) was modelled by Fourier's Law (Equation 6), where K is the thermal conductivity (W.m^−1^.K^−1^) and 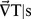 the temperature gradient (K.m^−1^) normal to the surface S. In the model, 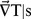 is approximated by the ratio of the temperature difference (ΔTemperature) to the distance (Distance) between the two cells adjacent to the surface S.
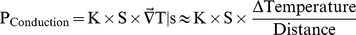
(6)


### Numerical method

#### Fruit mesh

Fruit volume was divided into sub-units by a regular mesh (Figure 1C). The increase in the number of sub-units increases the precision of the model predictions, but increases the calculation time as well. A mesh with more than 800 sub-units was considered to be a good compromise. The physical and thermal properties of sub-units were defined according to their position in the fruit and the tissue that they belonged to (Figure 1B).

### Determining thermal conductivity

Since thermal conductivity was considered to vary within the fruit, calculation of K between two sub-units with different thermal properties was done using Equation 7, where K is the resulting conduction coefficient (W.m^−1^.K^−1^) between two sub-units separated by the sum of the distances from the centre of each unit to the interface of the two sub-units (e_1_ and e_2_, in m), and K_1_ and K_2_ are the thermal conductivity (W.m^−1^.K^−1^) of the two sub-units, respectively.
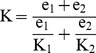
(7)


The total heat flux by conduction received by a sub-unit of the fruit is equal to the sum of the conduction exchanges between all adjacent sub-units.

#### Equation resolution

The equation system was solved using the finite volume method with an explicit method for time integration. A Courant-Friedrichs-Lewy (CFL) condition [41] was therefore used to ensure the stability of the numerical integration, leading to a time step Δt that was smaller than the critical CFL number, defined as 
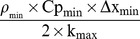
 where Cp_min_ is the minimum heat capacity (J.Kg^−1^.K^−1^) of the sub-units, Δx_min_(m) is the minimum distance between two sub-units, ρ_min_ is the minimum sub-unit density, and k_max_ is the maximum thermal conductivity of the sub-units.

### Fruit material, measurements and model parameterisation

#### Fruit material

The study was carried out at the CIRAD Research Station located in St. Pierre (Reunion Island, 20° 52′ 48″ S, 55° 31′ 48″ E), with CIRAD's permission. It was conducted on 18-year-old (in 2008) mango trees, Mangifera indica L. cv. “Cogshall” grafted on “Maison rouge”. Trees were well irrigated, spaced 5×6 m, and were approximately 3 m high. Measurements of surface temperature and of internal factors were taken on fruits of the 2006 and 2008 growing seasons, respectively. Four maturity stages were distinguished on the basis of the number of Days After full Bloom (DAB). The first stage, M1, corresponded to an immature green fruit with a size of approximately 6–8 cm, which corresponded to the end of cell division (DAB = 60). The second stage, M2, made reference to a green immature fruit in the cell expansion phase (DAB = 90). The following maturity stage, M3, described a green mature stage (DAB = 120). The last stage, M4, described mature fruits (DAB = 130).

### Measurements of climatic variables and fruit surface temperatures

Climatic data such as air temperature, global radiation, air humidity and wind speed were measured every minute and averaged and stored every hour on a data logger (Model 21 X, Campbell Scientific Ltd.; Logan, UT, USA) during the 2006 and the 2008 growing seasons. Direct and diffuse parts of global radiation were estimated using the model developed by Maxwell [42]. The sun's course was determined using the solar position algorithm (SPA) developed by Reda and Andreas [43]. The fraction of the global radiation received by the shaded side of a fruit located at the exterior of the canopy was equal to 20% of the global radiation, according to our measurements.

Fruit surface temperature measurements were carried out on three green immature, three green mature and three mature fruits during the 2006 growing season. These fruits corresponded to well-exposed fruits at the M2, M3 and M4 maturity stages, measuring approximately 115, 123 and 125 mm in length, 73, 78 and 80 mm in large diameter, 69, 73, and 75 mm in small diameter, and weighing approximately 269, 330 and 350×10^−3^ kg, respectively. Copper-constantan thermocouples (diameter: 0.2 mm) were attached to the fruit surface on the sunny and shaded sides. Measurements of fruit temperature surface were taken every minute and averaged and stored every hour on a data logger (Model 21 X, Campbell Scientific Ltd.; Logan, UT, USA) over three (from 28^th^ to 30^th^ December 2006), eight (from 12^th^ to 19^th^ January 2007) and three days (from 22^nd^ to 24^th^ January 2007) for fruits at the M2, M3 and M4 maturity stages, respectively.

#### Measurements of physical properties

Three model parameters related to radiation must be known before simulations can be carried out: fruit surface reflectance to short-wave radiations (A_sw_), fruit surface reflectance to long-wave radiations (A_lw_), and the emissivity for long-wave radiations (ε). No data was available in the literature for mango peel emissivity, so it was considered to be equal to peach surface emissivity, ε = 0.94 [20]. The A_lw_ value was deduced from the fruit surface emissivity using Krichhoff's law: Alw  = 1−ε.

Fruit peel reflectance (A_sw_) to short waves of solar irradiation was calculated using Equation 8, where the peel reflectance spectra was determined on 10 to 20 fruits for each fruit side and each maturity stage, from 350 nm to 2500 nm, every 1 nm, with a portable spectrometer (LABSPEC 2500, Analytical Spectral Devices, Inc.; Boulder, CO, USA), and the sun spectral irradiance values were taken from ASTM G173-03 standard tables established by the American Society for Testing and Materials (ASTM).
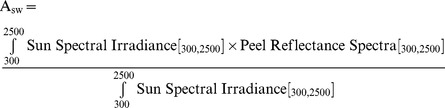
(8)


Fruit conductance (Gw, in m s^−1^) was calculated for sunny and shaded fruit, on 6 to 20 fruits for each maturity stage, using the method detailed by Léchaudel et al. [25].

The model simulates the increase in fruit size by considering a constant number of sub-units and by increasing each sub-unit's dimensions by a proportion of the fruit growth rate that has to be determined prior to the simulation. It was assumed that fruit proportions were constant during fruit development, so the fruit growth rate was determined on the basis of the increase in fruit height, calculated from the average of ten fruits measured weekly from the end of cell division (60 DAB) to total fruit maturity (130 DAB). Stone dimensions were deduced from fruit dimensions according to the following empirical relationships:
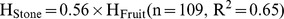


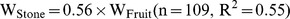


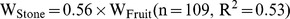
where W_stone_ and W_stone_ are the large and small diameters of the stone, respectively.

Peel thickness was considered constant and equal to 1 mm for Cogshall mango.

#### Estimation of fruit thermal properties

Fruit thermal properties such as heat capacity (Cp, in J.Kg^−1^.K^−1^) and thermal conductivity (K, in W.m^−1^.K^−1^) vary according to water content. The relationship given by Valente and Nicolas [44] was used to estimate thermal conductivity (Equation 9). Concerning the heat capacity, the relationship given by Siebel [45] was used (Equation 10).
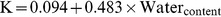
(9)

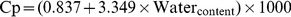
(10)


The water content of each compartment was deduced from equations taken from Léchaudel et al. [30] and the fruit fresh mass was deduced from the growth measurements on the ten fruits. Since mesh resolution was higher than the peel thickness, sub-units located at the fruit surface were a mix of peel and pulp tissues. Thus, heat capacity and thermal conductivity of these sub-units were determined according to the relative proportion of pulp and peel.

The specific gravity of fruit and stone was measured on 8 to 12 fruits at each of the four maturity stages. Specific gravity was calculated using Archimedes' principle, by measuring the fruit fresh mass in the air and its upward force when the fruit was immersed in water with a basket hanging from a balance. The specific gravity (Sg, Kg.m^−3^) is deduced as 
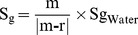
, where m is the mass (kg) of the fruit or the stone, r is the upward force (kg), and Sg_water_ is the specific gravity of water in which the fruit is immersed, considered as a constant (Sg_water_  = 1000 Kg.m^−3^). For the sake of simplicity, the specific gravity of peel and pulp were assumed to be equal and was deduced from the specific gravities of the fruit and stone.

### Measurements of peel colour

XYZ coordinates of peel colour were calculated from the peel reflectance values (see section: Measurements of physical properties) and converted to CIELAB coordinates. Since they have been described as good colour descriptors of fruit [32], [46], hue angle (H°) and chroma (C) values were calculated from a and b coordinates (Equation 11 and Equation 12, respectively). 
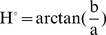
(11)

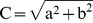
(12)


### Simulations and post-processing

Fruit temperature and transpiration were simulated from the phase of cell expansion, which begins after the period of cell division when the mango reaches approximately 6–8 cm in length [47], to ripening. Fruit internal factor changes according to the number of DAB were given as inputs into the model in order to simulate fruit temperature and transpiration during fruit development.

### Model assessment

As proposed by Saudreau et al. [20], the ability of the model to simulate heat conduction within a material and to handle heat fluxes at boundary conditions was first tested by simulating a physical situation for which an analytical solution exists. The case of a spherical object of radius R = 4×10^−3^ m, initially at a temperature T_0_ = 16°C, immersed in an atmosphere at a constant temperature T_a_ = 20°C, was studied. The sphere was heated by a constant convective heat transfer coefficient h = 5 W.m^−2^.K^−1^ and had a heat capacity of 3600 J.Kg^−1^ K^−1^ and a thermal diffusivity of 0.1 W.m^−1^.K^−1^. The expression of the analytical solution is detailed by Saudreau et al. [20].

Secondly, the model was assessed in a more realistic and complex situation by simulating the temperature dynamics over several days at three maturity stages of the fruit, i.e., M2, M3 and M4, during which temperature measurements were taken. For this case, input parameters and input climatic data were those relative to the measurement period.

### Analysis of spatial and temporal variations of energy exchanges, temperature and transpiration

Simulation outputs carried out on fruits throughout the day-night cycle from 12^th^ January 2007 at the four studied maturity stages with climatic input data were analysed in detail. Variations of power (in W.kg^−1^) by radiation, convection, transpiration and conduction at different locations within the fruit - at the fruit centre and at the surface of the shaded and the sunny sides - were compared. In addition, the variability of temperature and transpiration during the day-night cycle and within the fruit was examined for fruits at the M1 and M3 maturity stages with climatic input data from 12^th^ January 2007.

### Model analysis

A one-at-a-time sensitivity analysis was performed with the same input data as those used for testing the model assessment in order to identify the most influential parameter on temperature and transpiration. Conceptually, the simplest method for carrying out a sensitivity analysis is to repeatedly vary one parameter at a time while keeping the others fixed [48]. As proposed by Génard and Souty [49], the sensitivity of the minimum, the mean and the maximum value of temperature and transpiration simulated for the whole fruit to changes in parameter values (±20%) was studied using sensitivity coefficients. A sensitivity coefficient is equal to the ratio of the change in output (minimum, mean or maximum predicted temperature and transpiration value), to the change in the input parameter (0.4), while all other parameters remain constant [48]. This local sensitivity analysis provides information about the effect of small changes in the parameters on the model responses and does not provide information about the effect of simultaneous or large parameter changes.

A second analysis was performed to evaluate the impact of variations in thermal and physical fruit properties during fruit development on its temperature and its transpiration, taking their measured evolutions into account. Temperature and transpiration simulations were carried out with the climatic data of the 2008 growing season and with the measured evolution of fruit properties as input data. The impact of the evolution of the fruit properties during fruit development was determined by simulating temperature and transpiration, fixing one fruit property at a time to its initial value, corresponding to the M1 maturity stage. The effect on the evolution of all parameters on fruit temperature and transpiration was studied by fixing all parameters at the values measured at the first maturity stage.

### Statistical analysis

All analyses were performed with R software [50], implemented with the Colorspace [51] and R.matlab [52] packages. For evaluating model accuracy, the Root Mean Square Error (RMSE) was calculated using Equation 13, where y_t_ is the t^th^ observed or reference value, 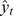 is the t^th^ simulated value, and n is the number of observed or simulated values. Multiple comparisions of measured fruit properties and peel colour descriptor averages were performed using the Tukey test. Graphical renderings were performed using Paraview software [53]. 
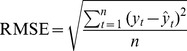
(13)


## Results

### Variations of the measured physical and thermal properties during fruit development

**Figure 2:**
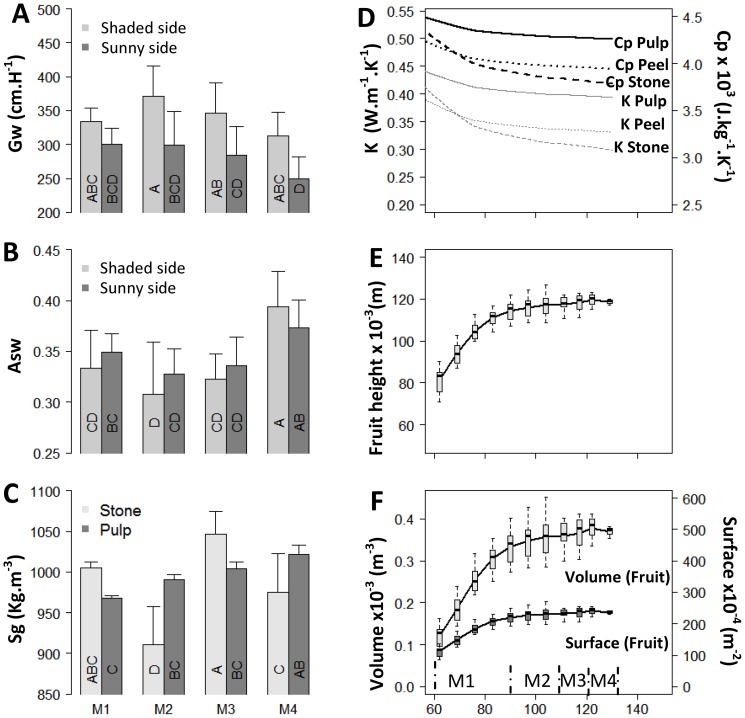
Thermal and physical parameters measured on mangoes, including peel conductance to water (A) and peel reflectance (B) according to four maturity stages and two sun exposures, the specific gravity of the stone and the pulp (C) according to four maturity stages, the heat capacity and the thermal conductivity of the stone, the pulp, and the peel (D), the fruit height (E) volume and surface (F) according to the number of days after full bloom. Different letters signify that the averages of the treatments are significantly different at P<0.05 (according to Tukey's multiple comparison test).

For all tested maturity stages except for the first one, peel conductance to water of sunny fruit was significantly lower than that of shaded fruit (Figure 2A). The maximum difference was found at the M2 maturity stage where the peel conductance to water of the sunny side was 19.3% lower than the shaded one. Peel conductance to water decreased for both fruit sides from M2 to M4, even if no significant difference could be established.

Hue angle (H°) and chroma (C) values of the peel of the sunny and shaded sides were found to be significantly different at all maturity stages except for the earliest one (Table 1). The shaded side remained green during fruit development, with hue angle values comprised between 116.6° and 122.7°, until the last development stage when the fruit was ripe. At this stage (M4), it turned yellow, with an H° value of 94.7° (Table 1). At the first stage of fruit development, the sunny side was green and turned red, as indicated by the H° values lower than 58.7°. Whereas colour varied significantly between fruit sides, no significant difference was found between the reflectance coefficients (A_sw_) of the sunny and the shaded sides (Figure 2B). Changes in the A_sw_ values during fruit development were similar for both fruit sides, with a continuous rise from the M2 maturity stage to the M4 stage. Maximal increases in reflectance values were observed between the M2 and M4 maturity stages, reaching 27.8% and 13.8% for the shaded and the sunny fruit sides, respectively.

**Table 1:** Effects of maturity stage and sun exposure on the hue angle, chroma and lightness of fruit peel.

		M1	M2	M3	M4
**Shaded side**	Hue angle	116.61 (A)	122.64 (A)	122.24 (A)	94.70 (B)
	Chroma	30.26 (bc)	33.19 (ab)	33.71 (ab)	37.86 (a)
	Lightness	52.8 (*b*)	49.73 (*b*)	51.03 (*b*)	67.5 (*a*)
**Sunny side**	Hue angle	113.66 (A)	58.68 (C)	9.31 (D)	20.18 (D)
	Chroma	30.08 (bc)	13.14 (d)	15.24 (d)	26.59 (c)
	Lightness	53.26 (*b*)	43.93 (*c*)	40.93 (*b*)	49.93 (*b*)

Different capital, lower and italic case letters signify that data are significantly different for the hue angle value, chroma and lightness, respectively, at P<0.05 (according to Tukey's multiple comparison test).

Significant differences were found between the specific gravities of stone and pulp, especially at the three maturity stages, M2, M3 and M4 (Figure 2C). The maximum difference was found at the M2 maturity stage where the specific gravity of the stone was 8.0% lower than that of the pulp. Specific gravities of stone and pulp significantly changed with fruit maturity stages, but their respective evolutions were different since the specific gravity of the pulp continuously increased, contrary to that of the stone, which was inconstant (Figure 2C). Maximal differences in stone density were observed between the M2 and M3 maturity stages since an increase of 14.8% was measured between these two maturity stages. For the specific gravity of the pulp, the greatest difference was measured between the first and the last maturity stages and represented an increase of 5.6%.

Specific gravity increased by 5.6% for the pulp and decreased by 2.9% for the stone from the first to the last maturity stages.

According to our estimation, based on Equation 9 and Equation 10, thermal properties such as heat capacity (Cp) and thermal conductivity (K) varied between the three fruit tissues - peel, pulp and stone - and decreased with fruit development (Figure 2D). During all fruit development stages for all fruit tissues, Cp and K decreased by less than 20%. Except for the first maturity stage, the Cp and K of the stone were lower than those of the peel, which were themselves lower than those of the pulp. The maximum difference was found at the M4 maturity stage where the Cp and K of the pulp were 15.31% and 16.13% higher than those of the stone, respectively. Fruit dimensions increased by 46.13% from the first maturity stage to the last one (Figure 2E), inducing an increase in the fruit volume and surface area (Figure 2F) of 208.31% and 112.61%, respectively.

### Model validation and spatial-temporal variations of temperature and transpiration

The temperatures predicted by the model for the case of a spherical object were close to analytical solutions (RMSE<0.041, R^2^ = 0.998; Figure S1) and proved that conduction and convection processes were well integrated into the model.

**Figure 3:**
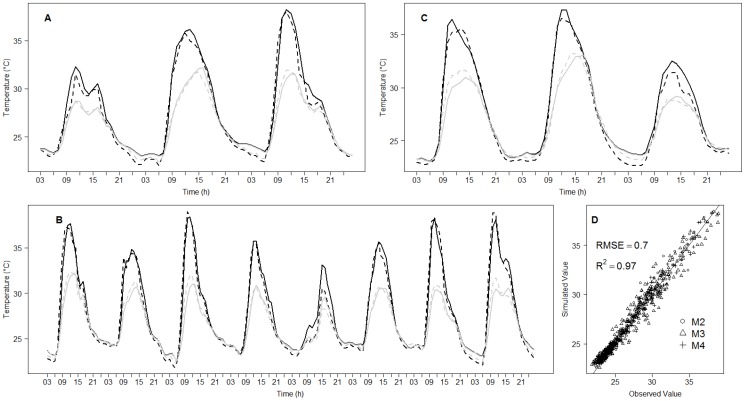
Changes in fruit surface temperatures for a period of 3, 8 and 3 days, measured (dashed line) and simulated (solid line) at the centre of the sunny side (black line) and at the centre of the shaded one (grey line) of a sunny fruit at the M2 (A), M3 (B), M4 (C) maturity stages, and comparison of these simulated data with those observed (D), with the curve y = x (continuous black line), the determinant coefficient, R^2^, and Root Mean Square Error (RMSE), between observed and predicted values.

Surface temperatures simulated by the model for the sunny and the shaded sides of a fruit were compared to the ones measured for a period of 3, 8 and 3 days for fruits at the M2, M3 and M4 maturity stages, respectively (Figures 3A to 3C). The model was able to accurately predict spatial and temporal temperature variations of mango fruit, regardless of the maturity stage, as shown by the good agreement with the measurements (R^2^>0.97 and RMSE = 0.7°C; Figure 3D).

**Figure 4:**
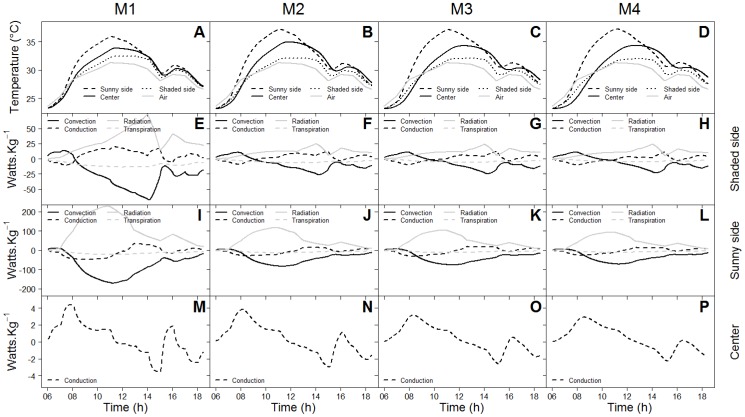
Daily changes in air and fruit temperatures at different positions within the fruit - the sunny side, the centre and the shaded side - at M1 (A), M2 (B), M3 (C) and M4 (D) maturity stages, and variations of power per mass unit by convection, conduction, radiation and transpiration of sub-units on the shaded fruit surface (E to H), on the sunny fruit surface (I to L) and at the fruit centre (M to P) for fruit at the M1, M2, M3 and M4 maturity stages.

Figure 4 compares the daily changes in simulated temperatures of sub-units located on the fruit surfaces of the sunny and shaded sides, and at the fruit centre, as well as the various components of the heat budget of these three fruit sub-units over one day for all of the maturity stages studied.

Differences in temperature between the sunny and the shaded sides of the fruit increased with fruit maturity stages (Figures 4A to 4D). The highest powers per mass unit were simulated at the M1 maturity stage (Figures 4E to 4P). Differences in power per mass unit between maturity stages were related to the variations of the physical and thermal fruit properties over time and to the more rapid increase in fruit volume than in fruit surface (see previous section). The major components of the energy balance of the fruit surface were the radiation and the convective rates (Figure 4E to 4L). The transpiration flux had the lowest impact on the heat budget of elements on the fruit surface and was negligible in comparison to the other heat fluxes (Figures 4E to 4L).

The influence of the radiation flux on fruit temperature was particularly evident around 3:00 pm when the low increase in radiation (Figures 4E to 4L) induced an increase in temperature at the different fruit positions (Figures 4A to 4D).

Powers simulated were greater for the sunny side of the fruit (Figures 4I to 4L) than the shaded one (Figures 4E to 4H), regardless of the maturity stage. For example, radiation and the convective powers were more than three times higher for the sunny side than for the shaded one at midday, regardless of the maturity stage.

The analysis of the conductive rate in the fruit centre indicated that during the first part of the day, the surface layers heat the underlying ones since the conductive heat is positive, whereas during the second part of the day, the underlying layers heat the surface layers since the conductive heat is negative (Figures 4M to 4P).

**Figure 5:**
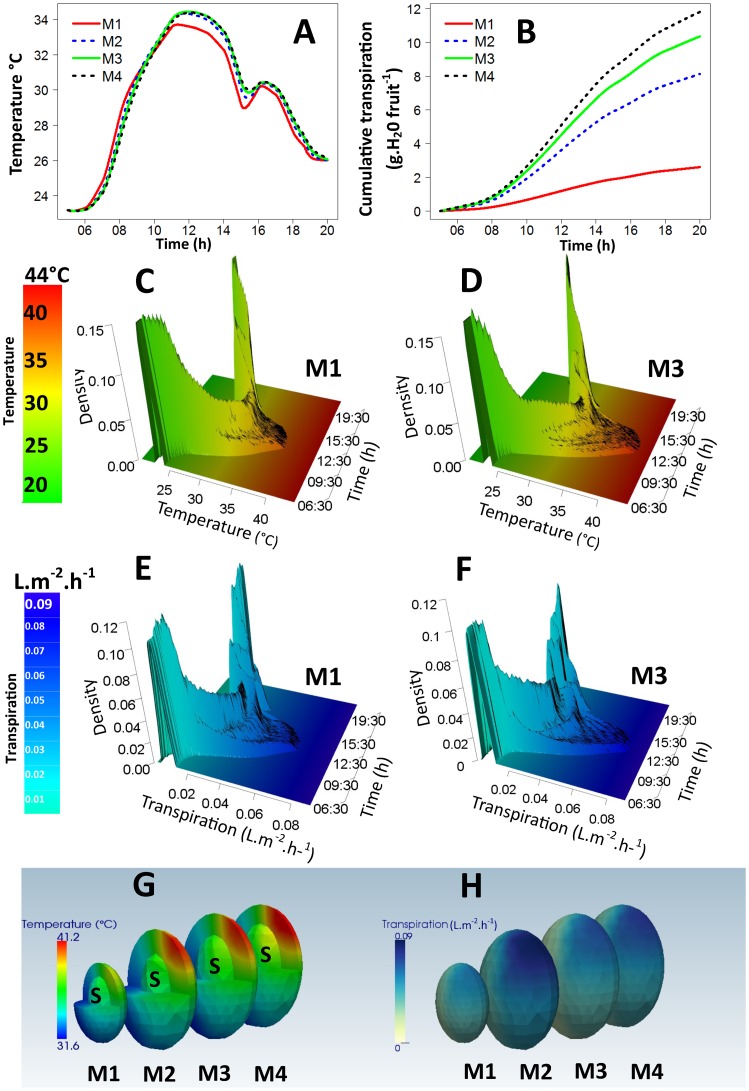
Effects of the maturity stage on the daily spatial and temporal variations of the temperature and the transpiration of fruit. The average daily evolution of temperatures (A) and cumulative water losses by transpiration (B) for fruits at the M1, M2, M3 and M4 maturity stages, and the probability distribution functions of the simulated temperatures (C and D) and the simulated transpiration rates (E and F) within the fruit at the M1 and M3 maturity stages, respectively, are represented. At a given time, the curve has a total area of one. The spatial distribution of temperatures (G) and water losses by transpiration (H) at 12:00 were obtained from simulations of a fruit at the four maturity stages, M1 to M4. S corresponded to the stone compartment.

Patterns of the diurnal changes in the average fruit temperature were quite similar, regardless of the maturity stage (Figure 5A). It was observed that the average fruit temperature of the smallest fruit, i.e., at the M1 maturity stage, increased and decreased faster than that of the large fruits, i.e., at the M2, M3 and M4 maturity stages. Daily water losses by transpiration increased with fruit maturity stages (Figure 5B). For fruit at the M4 maturity stage, the daily sum of water losses by transpiration reached 11.81 grams and represented more than 4.5 times that of a fruit at the M1 maturity stage.

The daily variability of the temperature (Figures 5C and 5D) and transpiration (Figures 5E and 5F) of fruits at the M1 and M3 stages were compared. Fruits at the M1 and M3 maturity stages were considered because they had contrasting sizes. Fruits start to grow at the M1 maturity stage, whereas they have almost reached their final size at the M3 maturity stage. The daily changes in the distributions of temperature and transpiration showed similar patterns for the two maturity stages. Temperature and transpiration were homogenous before the sun rose, and became more and more variable until the middle of the day (Figures 5C to 5F). Then, as the sun went down, fruit temperature and transpiration tended to return to the homogenous state observed at the beginning of the day. Temperature and transpiration gradients were more pronounced for the oldest fruit since they increased faster in the morning and decreased more slowly in the afternoon (Figures 5C to 5F). The gradients of temperature and transpiration were found to be approximately 9.5°C (Figure 5D) and 0.051 L.m^−2^.H^−1^ (Figure 5F) for the oldest fruit, compared to 6.8°C (Figure 5C) and 0.036 L.m^−2^.H^−1^ (Figure 5E) for the youngest one. These gradients are illustrated in Figures 5G and 5H, which compare the spatial variability of temperature and transpiration at 12:00 pm of fruits at the M1, M2, M3 and M4 stages. Temperature gradients within fruits at the M2, M3 and M4 maturity stages were similar and higher than those within fruit at the M1 maturity stage. The highest transpiration gradient was simulated for fruit at the M2 maturity stage and can be related to the highest difference in peel conductance to water between sunny and the shaded fruit sides measured for this maturity stage (Figure 2A).

### Sensitivity analysis to fruit internal factors and impacts of their measured evolution on fruit temperature and transpiration

The effects of variations of thermal and physical fruit properties on fruit temperature and transpiration were studied for different positions within the fruit - at the fruit centre and at the surface of the sunny and shaded fruit sides - using the sensibility coefficients calculated with the average, maximum and minimum predicted values. For the sake of simplicity, only the results related to the maximum values are presented since they showed the highest sensitivity to parameter variations (Table 2). Predicted temperature and transpiration values were slightly affected by the parameter variations, with the exception of the impact of the G_w_ variation on transpiration. Temperature and transpiration of the sunny side were found to be more sensitive to parameter variations than those of the other positions within the fruit. Peel reflectance, A_sw_, and fruit size were the model parameters that had the most impact on fruit temperature for all tested positions within the fruit. Skin permeability to water, G_w_, and peel reflectance, to a lesser extent, were found to have the highest impact on fruit transpiration for both fruit sides.

**Table 2:** Simple analysis of the sensitivity of simulated temperature and transpiration to parameter variations, based on the sensitivity coefficient of the maximum value.

	Centre_T°max_	Sun_T°max_	Shade_T°max_	Sun_Transpiration max_	Shade_Transpiration max_
**Heat Capacity (Cp)**	−6.4E-03	−1.3E-02	−9.9E-04	−3.3E-03	−3.8E-04
**Reflectance (Asw)**	−5.6E-02	−1.3E-01	−2.4E-02	−5.3E-02	−1.2E-02
**Specific gravity**	−6.4E-03	−1.3E-02	−9.9E-04	−3.3E-03	−3.8E-04
**Skin permeability to water (Gw)**	−2.2E-03	−2.3E-03	−2.4E-03	1.0E+00	1.0E+00
**Thermal conductivity (K)**	8.6E-04	−3.8E-02	1.4E-02	−1.1E-02	6.8E-03
**Peel thickness**	3.5E-05	1.4E-04	−1.4E-05	-8.3E-05	8.5E-05
**Stone/Fruit ratio**	2.2E-03	3.2E-02	−1.7E-02	1.0E-02	−8.2E-03
**Fruit size**	2.8E-02	6.2E-02	6.3E-03	1.4E-02	2.5E-03

A value of 1 or −1 signifies that the variations of the parameters induce a proportional increase or decrease, respectively, of the maximum of simulated temperature or transpiration.

Table 3 summarizes the effects of the evolution of physical and thermal parameters during a fruit's growth on its temperature and transpiration. The greatest effect of the evolution of fruit properties was found for the total water losses by transpiration since these losses were multiplied by more than 2.8 when taking parameter variations into account. Evolutions of fruit properties had minor effects on the minimum and the average of predicted temperatures, whereas the predicted maximum values of temperature and transpiration per surface unit somewhat increased. The impact of the evolution of a fruit's characteristics in terms of transpiration and temperature were mainly due to its increase in size. The evolution of the peel conductance to water did not sharply affect fruit transpiration. The reflectance evolution did not affect fruit temperatures.

**Table 3:** Effects of the variation of thermal and physical parameters during fruit development on fruit temperature, maximum transpiration per surface unit and total water losses by transpiration.

	Temperature mean (°C)	Temperature max (°C)	Temperature min (°C)	Transpiration max (L.m^−2^.H^−1^)	Total water losses (L)
**Evolution of all parameters**	24.20	41.38	17.00	7.241×10^−2^	0.569
**All parameters constant**	24.18	39.27	16.99	6.604×10^−2^	0.202
**Constant fruit size**	24.19	39.27	16.99	6.607×10^−2^	0.191
**Constant fruit conductance to water (Gw)**	24.21	41.37	17.00	7.360×10^−2^	0.547
**Constant fruit reflectance (Asw)**	24.18	41.00	17.04	7.081×10^−2^	0.567
**Constant thermal parameters (K, Cp, SG)**	24.20	41.21	17.00	7.161×10^−2^	0.569

## Discussion

### Model accuracy and gradient of transpiration and temperature within the fruit

The established model in this study is able to predict fruit temperature variations at both space and time scales, taking changes in physical and thermal parameters during fruit development and between fruit tissues into account. The discrepancy between the model predictions and the observed data was satisfactory and on the same order of magnitude of another fruit temperature model previously developed by Saudreau et al. [20]. Confirming the previous study of Saudreau et al. [20], radiation and convection were the main heat fluxes driving fruit temperature. The fruit temperature surface area was found to differ from that of the air and to greatly vary during the day, as observed for many fruits such as apple [20], [54], peach [20] and avocado [55]. The simulated gradient of temperature and transpiration within the fruit fluctuated during the course of the day and was non-negligible since it exceeded 9°C and 0.051 L.m^−2^.h^−1^, respectively. The large inner temperature gradient previously observed by Saudreau et al. [20] and the large transpiration gradient should be considered for modelling physiological processes. It was recently suggested that differences in light conditions between the two sides of a mango induce the differences in water and osmotic potentials observed between the sunny and shaded sides of the fruit [25]. Since temperature is known to affect enzymatic reaction speed, the temperature gradient is expected to induce differences in substrate and product concentrations within the fruit. Consequently, it would be interesting to determine whether or not the temperature gradient highlighted in this study is related to sugar variations within the fruit, in accordance with relationships established between the accumulation of temperature and sugar content for peach [49] and mango [2]. In addition, due to the impact of temperature on respiration rate [5]–[7], temperature variations within the fruit are expected to affect the gas concentration gradients previously reported for several fruits such as apple [56], [57] and pear [58]. Further studies are also required to study the impact of the transpiration gradient within the fruit highlighted in this study on mineral and water content. Water losses by transpiration induce variations in mineral content, as shown for several fruits such as kiwi [15] and apricot [59], and affect water content, as shown for tomato [14].

### Effects of variations of thermal and physical fruit properties during fruit growth on temperature and transpiration

None of the variations in fruit properties except those representing the increase in fruit size was found to have a high incidence on fruit temperature and transpiration. The absence of strong effects was due to the weak influence of fruit thermal and physical properties on fruit temperature and transpiration per surface unit, as revealed in the local sensitivity analysis, and to the fact that these properties changed very little during fruit development, as shown by their measurements. Although changes in fruit colour were observed during fruit development and according to the fruit side, peel reflectance was found to increase only slightly during the last maturity stage. The increase in peel reflectance during fruit ripening could be related to the degradation of chlorophyll pigments, which induces the appearance of yellow peel colour [60], as observed for the shaded side in the late development stage. Surprisingly, no difference in the peel reflectance values was found between the two fruit sides, although the sunny fruit side had a pink blush. A purple peel colour was effectively presumed to be related to a higher heat absorbing capacity than a green one [34]. In fact, the accumulation of anthocyanin pigments is reported to be involved in peel blushing [61], [62] in response to high irradiance [63], and is probably responsible for decreasing peel reflectance. As observed by Ribero Da Luz [64] for leaves, accumulation of wax on exposed tissues affects optical properties. Therefore, differences in wax accumulation between fruit sides due to sun exposure differences, as observed on grape [65], [66], could explain the absence of differences in reflectance values between the two fruit sides, even if their anthocyanin pigment contents were assumed to be different. Low variations in the peel conductance to water were found with the fruit maturity stages as well. The slight tendency of a decrease in the peel permeability to water with fruit development, regardless of the fruit side, could be explained by the assumed increase in the epicuticular wax per unit skin area [67], [68] and/or in the wound healing activity around cracks [26], [69]. The measured lower peel conductance to water of the sunny side compared to the shaded one would result from the presumably higher accumulation of wax on this side [65], [66]. Finally, the decrease in the water content during fruit growth [30] induced only a slight variation in thermal parameters, including heat capacity, thermal conductivity and specific gravity. Only the fruit size increase was found to extend the gradient of fruit temperature and transpiration, mainly due to the expansion in volume.

## Concluding Remarks

Significant variations in thermal and physical properties were measured between mango tissues at several mango maturity stages. To study the impact that mango development has on its temperature and transpiration, a physical model was developed. Model predictions have been satisfactorily compared to fruit temperature and have made it possible to accurately simulate the daily variations of temperature between fruit sides. Model outputs indicated that the average fruit temperature at the growing season scale was not affected by the evolution of the fruit's thermal and physical properties during its development. These results suggested that the calculation of an average temperature of a growing fruit, required, for example, for the calculation of the thermal time sum, can be achieved without taking the evolution of fruit properties into account. However, results indicated that changes in a fruit's properties during its development induced changes in the temperature and transpiration distribution within the fruit. Consequently, to represent the spatial and temporal variability of fruit temperature and transpiration of a growing fruit to model physiological processes such as growth, ripening and quality development, the variations in the fruit's properties during its development, and particularly its size, should be taken into account.

## Supporting Information

Figure S1 **Temperature variations simulated by the model and determined by an analytical solution of a spherical object at an initial temperature of 16°C, immersed in an atmosphere at a constant temperature of 20°C, at the sphere surface, i.e., r = R, and at the sphere center, i.e., r = 0, vs. time; expressed as the ratio of the time (T) to T0, the characteristic time at which the entire object reaches the air temperature.**
(TIF)
