# Correction: Model-Assisted Analysis of Spatial and Temporal Variations in Fruit Temperature and Transpiration Highlighting the Role of Fruit Development

**DOI:** 10.1371/journal.pone.0151196

**Published:** 2016-03-03

**Authors:** Thibault Nordey, Mathieu Léchaudel, Marc Saudreau, Jacques Joas, Michel Génard

In the Materials and Methods section, there is an error in the first equation. Please view the complete, correct equation here:

$$\Delta T=\;\frac{\Delta Q}{Sg\times \;V\;\times \;Cp}$$
