# Supplementary figures and images for: Model-Assisted Analysis of Spatial and Temporal Variations in Fruit Temperature and Transpiration Highlighting the Role of Fruit Development
Source: PLoS One. 2014 Mar 24;9(3):e92532. doi: 10.1371/journal.pone.0092532 (PMC3963907; doi:10.1371/journal.pone.0092532)

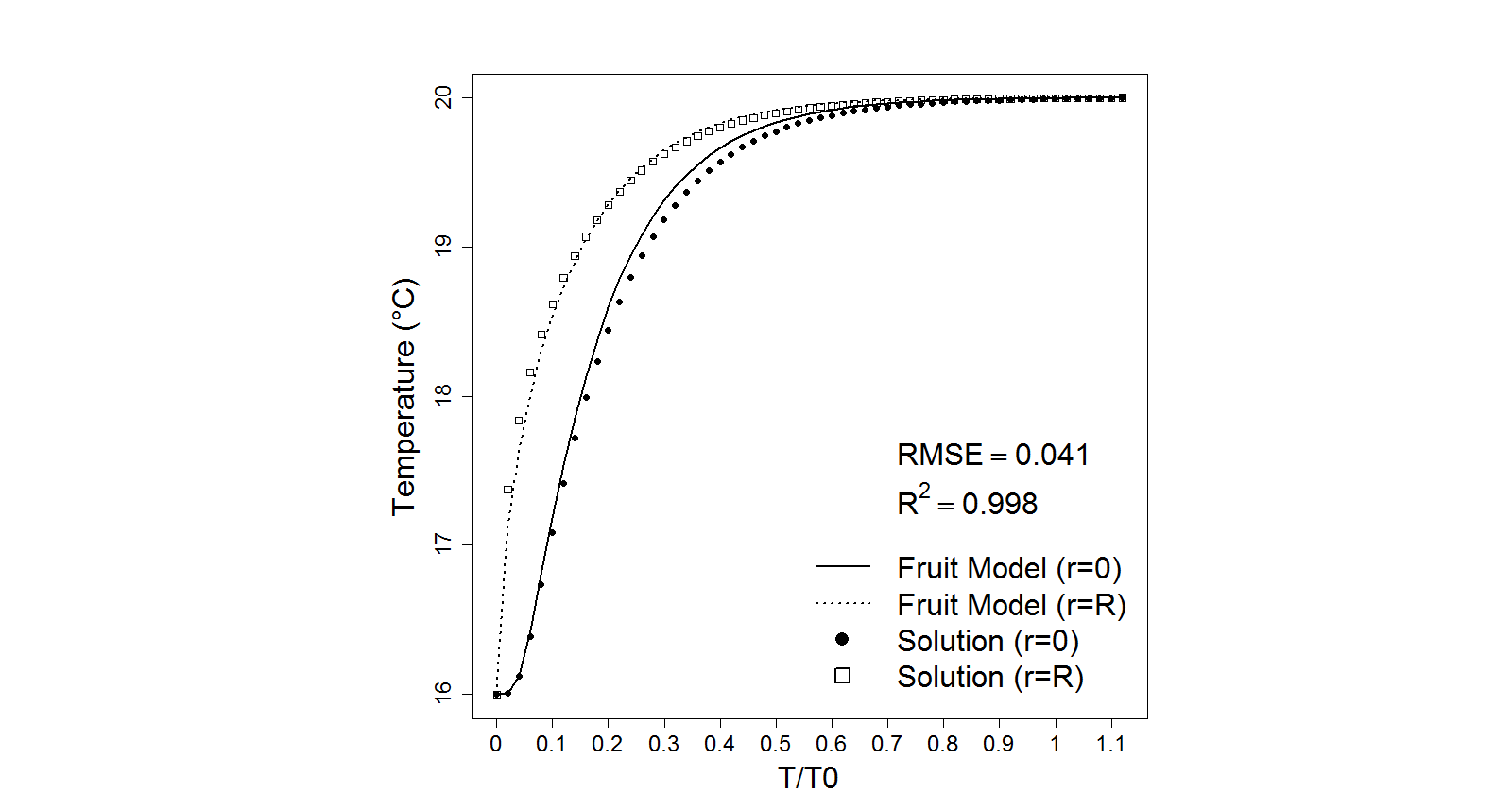

Supplement: Figure S1 — Temperature variations simulated by the model and determined by an analytical solution of a spherical object at an initial temperature of 16°C, immersed in an atmosphere at a constant temperature of 20°C, at the sphere surface, i.e., r = R, and at the sphere center, i.e., r = 0, vs. time; expressed as the ratio of the time (T) to T0, the characteristic time at which the entire object reaches the air temperature. (TIF) [file pone.0092532.s001.tif]
